# Recreational substance use and aneurysmal subarachnoid hemorrhage: differential effects of alcohol and THC

**DOI:** 10.1007/s10143-026-04295-w

**Published:** 2026-04-23

**Authors:** Marvin Darkwah Oppong, Annika Witte, Philipp Dammann, Argtim Rexhepi, Yahya Ahmadipour, Meltem Gümüs, Yan Li, Thiemo Florin Dinger, Laurèl Rauschenbach, Cornelius Deuschl, Džiugas Meška, Ulrich Sure, Ramazan Jabbarli

**Affiliations:** 1 Department of Neurosurgery and Spine Surgery, University Hospital Essen, University of Duisburg-Essen, Essen, Germany; 2 Center for Translational Neuro- & Behavioral Sciences (C-TNBS), University of Duisburg, Essen, Germany; 3 Department of Diagnostic and Interventional Radiology and Neuroradiology, University Hospital Essen, University of Duisburg-Essen, Essen, Germany

**Keywords:** Intracranial aneurysm, Aneurysmal subarachnoid hemorrhage, Alcohol, THC, Recreational drugs

## Abstract

Objective: The use of recreational drugs such as alcohol and tetrahydrocannabinol(THC) is increasing worldwide. While tobacco and certain illicit substances are well-established risk factors for aneurysmal subarachnoid hemorrhage (aSAH), the role of alcohol and THC remains less well defined. Understanding their potential impact on aneurysm rupture and clinical severity could have significant implications for prevention and patient management. This study aimed to investigate the influence of alcohol and THC use on the risk of intracranial aneurysm(IA) rupture and the clinical severity of aSAH. Methods: We prospectively included 954 patients with IA treated at a tertiary center in Germany between 2016 and 2023. Alcohol and drug use were documented through structured interviews. Risky alcohol use was defined as >20g/day(males) and >10g/day (females). Clinical severity of aSAH was assessed using the World Federation of Neurological Surgeons(WFNS) scale; radiographic severity was classified using the modified Fisher scale. Univariate and multivariate analyses were performed to evaluate associations between substance use, IA rupture, and severity. Results: Risky alcohol consumption was reported in 4.6% of patients, THC use in 5.3%, and polytoxicomania use in 3.2%. Risky alcohol consumption was independently significantly associated with IA rupture (adjusted odds ratio[aOR]2.00,95%,CI 1.07–3.75,p=0.031) and clinically severe aSAH (WFNS grade IV-V:aOR 3.26,95%,CI 1.34–7.95,p=0.009). No significant associations were observed for THC or polytoxicomania. Conclusions: Risky alcohol consumption was independently associated with the risk of IA rupture and more severe clinical presentation of aSAH. THC use showed no significant effect. Alcohol use should be actively addressed in IA patient counseling, and further studies are warranted to clarify the role of THC.

**Supplementary Information:**

The online version contains supplementary material available at 10.1007/s10143-026-04295-w.

## Introduction

The use of recreational drugs is widespread, with alcohol and tobacco being legal in most countries [1, 2]. In recent years, tetrahydrocannabinol (THC) has also been legalized in several nations [3]. It is widely known that the use of these substances can have harmful effects on general health. Regarding aneurysmal subarachnoid hemorrhage (aSAH), tobacco use has been established as one of the most significant risk factors for both the formation and rupture of intracranial aneurysms (IA) [4–6]. Additionally, the consumption of certain illicit drugs, such as cocaine and amphetamines, has been strongly linked to an increased risk of occurrence, rupture, and severity of rupture in IA [7–9].

Despite the wealth of research on tobacco and illicit drugs, the impact of alcohol on intracranial aneurysms remains underexplored. Recent studies investigating the relationship between alcohol consumption and the risk of rupture or severity of aSAH are limited. Previous studies oppose relevant deficits in qualities as reported in meta-analysis [10, 11]. However, an excessive alcohol consumption has been identified as a key risk factor [10, 12]. Meanwhile, the effect of THC use on these outcomes is even less understood [9, 13–16], as there is a notable lack of relevant studies in this area.

Given the growing prevalence of recreational THC use and the potential for alcohol to influence vascular health, there is a pressing need for further research. Understanding the role of alcohol and THC in the context of IA rupture could have significant implications for public health and clinical practice. This study aims to address this gap in knowledge by investigating the effects of alcohol and THC use on the risk and severity of IA rupture in cases of aSAH.

## Methods

All patients aged 18 years or older who were treated for an IA (ruptured or unruptured) between July 2016 and October 2023 at a single tertiary hospital in Germany were eligible for this study. The study received approval from the Institutional Review Board (Ethik-Kommission, Medizinische Fakultät der Universität Duisburg-Essen; Approval No. 15-6331-BO) and was registered with the German Clinical Trials Register (DRKS; ID DRKS00008749; Registration date: 06/09/2015). Patients were prospectively included in this study. After obtaining written consent from the patient or their next of kin, all clinical and radiographic data assessed during each visit were stored in the institutional ARCTICA (Assessment of Risk Clusters in Treatment of Individuals with Cerebral Aneurysms) database. Additional clinical information was collected via a paper-based interview with the patient or their next of kin at first presentation. Furthermore, a telephone-based interview was conducted at the 6-month follow-up to fill in any missing information and reconfirm the initial documentation. All patients and relatives were informed about the confidentiality of the obtained information.

Patients presenting with clinical signs of aSAH underwent cranial computed tomography (CT) to confirm aneurysm rupture. Patients suspected for aSAH with a normal CT scan received a lumbar puncture to rule out or confirm aSAH. The clinical status of aSAH patients was assessed using the World Federation of Neurosurgical Societies (WFNS) scale [17]. WFNS grades 4 and 5 were classified as poor-grade aSAH. The radiographic severity was assessed using the modified Fisher (mFisher) scale [18]. mFisher grades 3 and 4 were defined as radiographic severe aSAH.

All patients underwent additional digital subtraction angiography (DSA) to confirm the presence of the IAs. In cases with signs of herniation requiring immediate surgical treatment upon admission, CT angiography (CTA) was performed as stand alone. IA size and morphology were determined using DSA, or CTA when DSA was not available. IAs with multiple lobes or daughter aneurysms were classified as irregular. The location of the IA was further stratified into anterior and posterior circulation. Patients with more than one IA were classified as multiple IA (MIA) bearers.

Information on alcohol use, premorbid conditions, drug use, and positive family history of IA was collected during the interview. A positive family history was defined as at least one first-degree relative with an IA. Premorbid conditions including hypertension were assed as part of the structured interview, documented diagnosis from primary care physicans and regular medication intake before first presentation. use was categorized into THC use and polytoxicomania to identify patients with heavy use of illicit drugs. Polytoxicomania was defined as the use of two or more recreational drugs other than alcohol. Alcohol use was further stratified at a consumption of over 20 g per day in male and over 10 g per day in female patients, classified as risky alcohol use. We used the mean consumption over the last 12 month.

Statistical analysis was performed using SPSS Version 25 for Windows. Univariate analysis was conducted to assess correlations between different alcohol and drug use and the radiographic characteristics of the IA, as well as the presence of ruptured IA in the whole IA cohort and clinical severity of aSAH among cases with ruptured IA. Dichotomous variables were evaluated using Chi-square tests or Fisher’s exact test for sample sizes less than five. The significance level was set at *p* < 0.05. Finally, a multivariate analysis was performed for significant correlations identified in the univariate analysis to assess the independent association of recreational drugs with the occurrence and clinical severity of aSAH. Adjustments were made for the common risk factors age, smoking, IA sac size, sex, arterial hypertension, and family history of IA. Missing data for covariants in the multivariant analyses were replaced using multiple implications.

Chat GPT 5 (Open AI, 2025) was used solely for help with text style/grammar corrections and figure design. The scientific content, statistical data analysis, and interpretation of the results were entirely performed and verified by the authors without the use.

## Results

The final cohort consisted of 954 patients with IA. Eight patients were excluded due to missing data on alcohol or drug consumption. The majority of patients were female (674; 70.6%). Over two-thirds of the cohort (641; 67.2%) reported consuming alcohol to some extent, while 4.6% (44 patients) reported risky levels of alcohol consumption. THC use was reported by 51 patients (5.3%), and polytoxicomania was documented in 31 patients (3.2%). A total of 394 patients (41.3%) presented with a ruptured IA. A comprehensive list of baseline characteristics is provided in Supplemental Table 1.

### Sex and age differences in substance use in the whole IA cohort

**Fig. 1:**
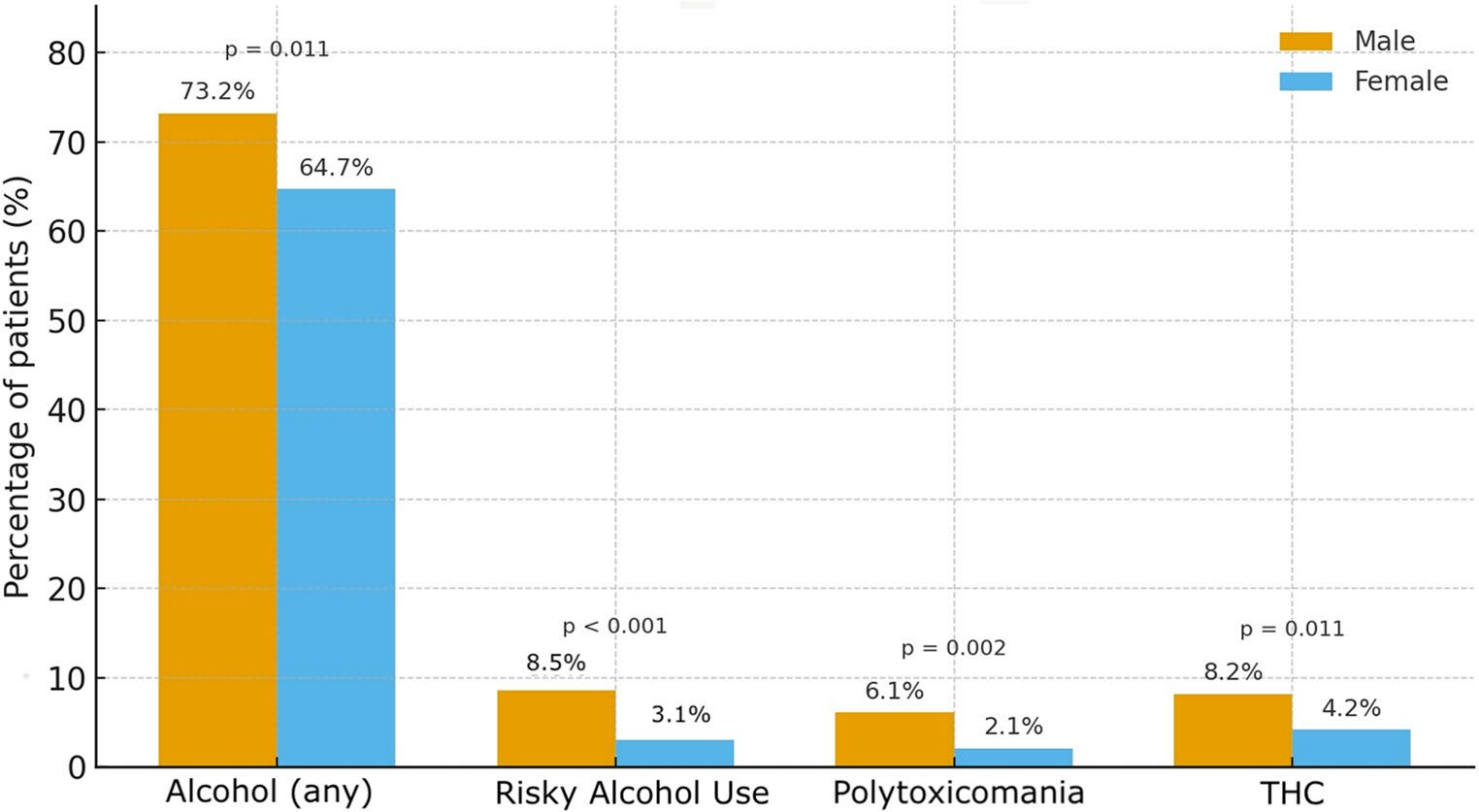
Different distribution of drug use by sex

For all categories of recreational drug use, consumption rates were significantly lower among female patients. Specifically, women were significantly less likely to consume any alcohol (*p* = 0.011; odds ratio [OR] 0.67; 95% confidence interval [CI] 0.49–0.91), engage in risky alcohol consumption (*p* < 0.001; OR 0.33; 95% CI 0.18–0.60), use THC (*p* = 0.011; OR 0.27; 95% CI not specified), or report polytoxicomania (*p* = 0.002; OR 0.33; 95% CI 0.16–0.68) compared to their male counterparts (Fig. 1).

Additionally, patients who used THC, consumed any alcohol or reported polytoxicomania use were significantly younger than those who did not. This age difference was not observed for risky alcohol consumption (Table 1).

**Table 1:** Mean age distribution between drug users and alcohol consumers

Parameter	Yes		No		*p*
	Mean age (years)	±SD (years)	Mean age (years)	±SD (years)	
Alcohol any	56	12	58	14	0.029
Risky Alcohol Use	54	11	57	13	0.256
THC	43	10	57	12	< 0.001
Polytoxicomania	42	10	57	13	< 0.001

### Impact of alcohol, THC, and polytoxicomania on IA rupture

Patients who reported risky alcohol consumption had a significantly increased risk of presenting with a ruptured IA in univariate analysis (*p* = 0.014; OR 2.13; 95% CI 1.15–3.94). This association remained significant after adjusting for common risk factors in multivariate analysis (*p* = 0.031; adjusted OR [aOR] 2.00; 95% CI 1.07–3.75; Table 2).

**Table 2:** Multivariant analysis of risk of IA rupture, significant parameters are marked bold

Parameter	*p*	aOR	95%CI
**Sex female**	**0.047**	**1.34**	**1.01–1.80**
**Age > 70 years**	**0.031**	**1.55**	**1.04–2.32**
Smoking	0.613	0.92	0.66–1.28
Art.hypertension	0.053	0.76	0.58–1.01
Familial IA	0.951	1.01	0.69–1.50
**IA sack>7 mm**	**< 0.001**	**1.68**	**1.28–2.21**
**Risky Alcohol Use**	**0.031**	**2.00**	**1.07–3.75**

No significant association with IA rupture was observed for general alcohol consumption (*p* = 0.751), THC use (*p* = 0.756), or polytoxicomania (*p* = 0.236) in univariate analysis.

### Impact of substance use on severity of aSAH

Risky alcohol consumption was associated with a significantly increased risk of presenting with clinically severe aSAH (*p* = 0.011; OR 2.95; 95% CI 1.24–6.90), but not with radiographically severe aSAH (*p* = 0.856; OR 0.93; 95% CI 0.40–2.14). In multivariate analysis, risky alcohol consumption remained an independently associated with clinically severe aSAH (*p* = 0.009; aOR 3.26; 95% CI 1.34–7.95; Table 3).

**Table 3:** Multivariant analysis of risk of presentation with a clinical severe aSAH (WFNS 4/5) in case of IA rupture, significant parameters are marked bold

Parameter	*p*	aOR	95%CI
Sex female	0.534	0.87	0.55–1.37
Age > 70 years	0.079	1.71	0.94–3.12
Smoking	0.437	0.81	0.48–1.38
**Art.hypertension**	**0.012**	**1.73**	**1.13–2.67**
Familial IA	0.119	0.59	0.31–1.15
**IA sack>7 mm**	**0.028**	**1.60**	**1.05–2.45**
**Risky Alcohol Use**	**0.009**	**3.26**	**1.34–7.95**

There was no significant association between any alcohol consumption, THC use, or polytoxicomania and either clinical or radiographic severity of aSAH (Table 4).

**Table 4:** Univariate analysis of risk of presentation with a clinical severe (WFNS 4/5) or radiographic severe (mFisher 3/4) aSAH in case of IA rupture

Parameter	WFNS 4/5				
	yes	no	*p*	OR	95%CI
Alcohol any	44.6%	46.5%	0.725	0.93	0.61–1.42
Polytoxicomania	50.0%	45.0%	0.692	1.22	0.45–3.33
THC	40.0%	45.5%	0.633	0.80	0.32-2.00
	mFisher 3/4				
	yes	no	p	OR	95%CI
Alcohol any	67.4%	71.7%	0.396	0.82	0.52–1.30
Polytoxicomania	68.8%	68.8%	> 0.99	0.99	0.34–2.93
THC	60.0%	69.3%	0.384	0.67	0.27–1.67

## Impact of substance use on aneurysm characteristics

**Fig. 2:**
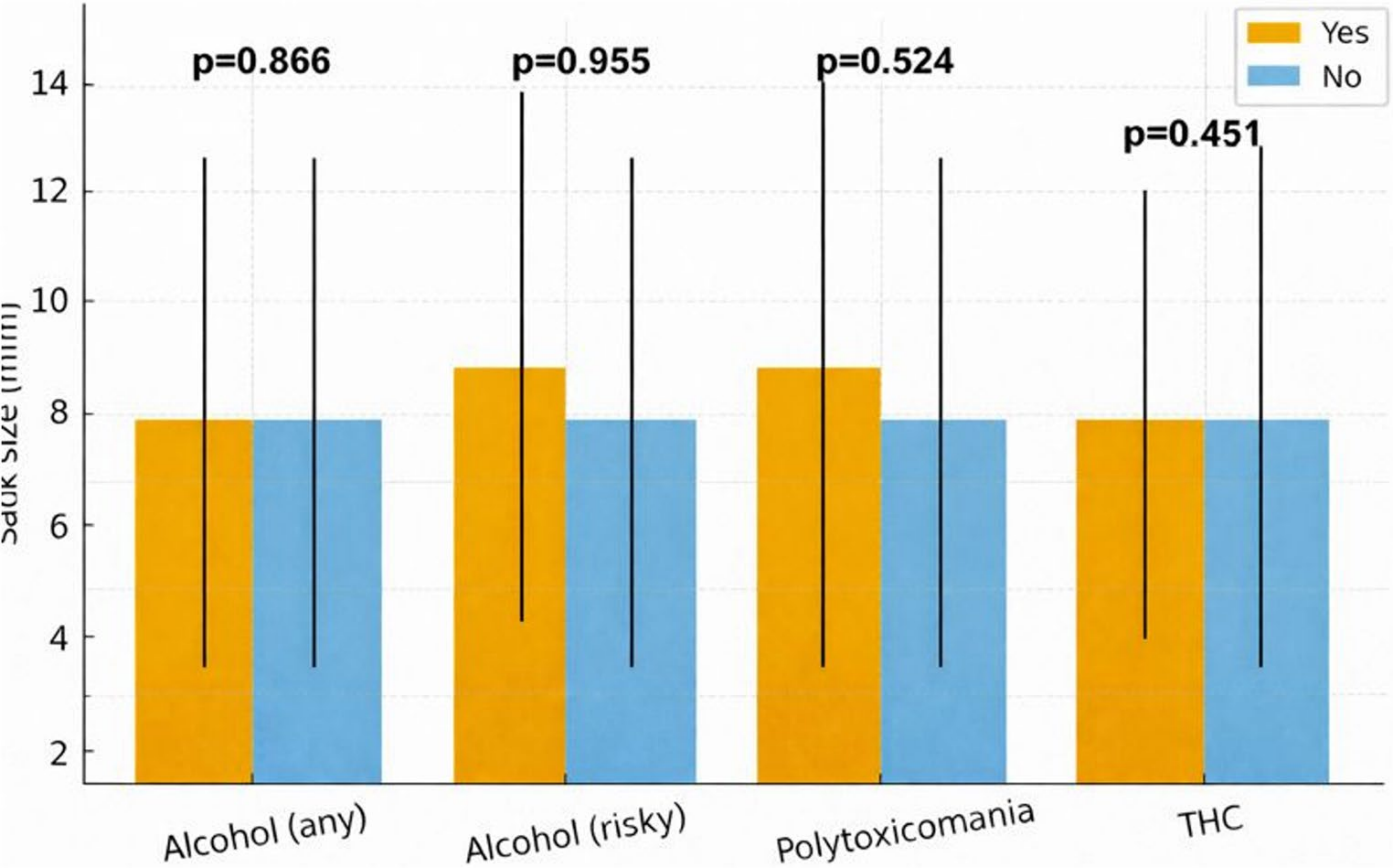
Differences in IA sack size depending on drug use

No significant differences could be shown regarding the size of the IA and the different drug uses (Fig. 2). Regarding the MIA patients with polytoxicomania showed the highest in group incidence with 48.4% and lowest rate of posterior circulation IA (6.5%). However, this difference showed no significant difference (*p* = 0.131 and *p* = 0.118, respectively). Furthermore, no subgroup showed a significant increased rate of irregular IA (Supplemental Table 2).

## Discussion

The aim of this study was to elucidate the influence of recreational drug use on the occurrence and severity of aSAH. We utilized our prospective IA database and included 954 patients over a six-year span. Alcohol consumption to any extent was relatively common, affecting about two thirds of the cohort. Risky alcohol consumption and THC use affected only a much smaller group of patients. Our data show a connection between risky alcohol consumption and IA rupture, as well as the clinical severity of aSAH. There were no corresponding results for patients consuming THC.

Alcohol is known as a substance with a multitude of harmful effects and is the origin of a relevant social burden. In addition to vascular diseases, it plays a role in multiple other chronic and malignant diseases [19, 20]. Previous studies have shown that current alcohol consumption and the amount of alcohol consumed increase the risk of aSAH, which is in line with our own results [10–12, 21–23]. Furthermore, three meta-analyses, mainly based on smaller and earlier studies with certain methodological limitations, drew a similar conclusion: they connected excessive alcohol consumption with IA rupture [10–12]. Other authors linked alcohol consumption to rupture and outcome, but only as part of a panel of factors contributing to an unhealthy lifestyle [24]. Additionally, genetically determined alcohol consumption has so far failed to predict IA rupture [23]. Our analysis also showed that risky alcohol consumption leads to clinically but not radiographic more severe aSAH. One possible explanation is that the systemic effects of alcohol—such as transient or chronic hypertension, impaired platelet function, and alcohol-related coagulopathy—may worsen early neurological status at presentation without necessarily increasing the initial hemorrhage volume. These mechanisms could contribute to a more severe clinical presentation independent of radiographic bleeding extent. Finally, patients with high levels of alcohol consumption often exhibit increased overall frailty and a poorer general health status. exact effect of alcohol on the risk of IA rupture is not fully understood. One possible mechanism may be that regular alcohol consumption increases the risk of hypertension [25]. However, in our study the negative effect of alcohol persisted even after adjusting for hypertension. Alcohol has been shown to cause oxidative stress [26] and leads to mitochondrial damage [27]. In turn, oxidative stress has been linked to IA formation and rupture [28]. Notably, it has been shown that in case of cessation of alcohol consumption the likelihood of IA rupture no longer increased, emphasizing the potential positive effects of alcohol cessation [21]. Acknowledging that alcohol consumption seems to play a crucial role only above certain levels, as demonstrated in our and other studies [10, 11, 21], it can be hypothesized that reducing consumption below a specific threshold might also have beneficial effects.

Results on the influence of THC on IA and aSAH are contradictory so far. There are three analyses of national cohort patient data regarding the use of THC in connection with IA. One reported no effect on outcome after aSAH but cited possible underreporting of usage [29]. Others reported a higher rate of perioperative complications in THC users with aSAH and a higher likelihood of aSAH occurrence [30]^,^ [14]. On the other hand, a retrospective single-institution analysis from 2022 found no association between THC consumption and aSAH occurrence [31]. Finally, a smaller study from 2006 found evidence of a negative effect of THC consumption on outcome after aSAH but failed to prove an independent association [13]. THC use has been associated with oxidative stress as well as with ischemic stroke in young adults. Both of these facts suggest possible negative effects on patients with IA [32, 33]. Our study failed to support these suggestions. One possible reason may be that the group of drug users in our cohort was relatively small and the heterogeneity regarding the amount and mode of consumption inside these groups was higher among THC users and patients with polytoxicomania than in the alcohol subgroup. Like in previous studies, we did not specify the method of THC consumption. As there are many different ways to consume THC, a possible effect of the route of administration remains unclear.

While multiple studies have connected the use of illicit drugs with the risk of aSAH [8, 9, 34–37], our study failed to draw a similar conclusion for patients consuming a mixture of such substances. The reasons for this appear to be multifactorial: one is the small sample size in our cohort, most likely due to underreporting and the fact that individuals with drug addiction tend not to undergo regular medical check-ups. Another theoretical explanation could be that patients with severe drug addiction may die from aSAH before reaching the hospital, which would also contribute to underreporting of such cases. We additionally failed, also most likely due to the small sample size, to connect drug use with different aneurysm characteristics.

## Limitations

This study faces several limitations. First, the study is single-center and not population-based. The number of THC users in this cohort is relatively small which limits the statistical power. One factor may be that the data were collected before THC use was legalized for recreational purposes in Germany (April 2024). Furthermore, it relies on self- or relative-reported drug and alcohol consumption which might be an additional reason for underreporting. Nevertheless, the relatively large sample size and the prospective data collection contribute to the robustness of this study.

## Conclusion

This prospective single-center study adds evidence to the growing body of literature supporting the negative effect of alcohol consumption on the risk of IA rupture, while also independently linking it to more severe clinical presentation in cases of aSAH. However, given the observational design, causality cannot be established. While no negative effect of THC use could be demonstrated in this study, this may be due to underreporting of polysubstance use. Alcohol consumption in patients with IA should be carefully evaluated and addressed in patient counseling regarding lifestyle modification after diagnosis. As an increasing number of countries move toward legalization of THC, further large-scale, multicenter studies with more detailed substance-use assessment will be necessary to better clarify the potential impact of THC and other psychoactive substances on IA progression and aSAH outcomes. 

## Supplementary Information

Below is the link to the electronic supplementary material.

Supplementary Material 1

## Data Availability

The data that support the findings of this study are not openly available due to reasons of sensitivity and are available from the corresponding author upon reasonable request.
