# Supplementary material for: Recreational substance use and aneurysmal subarachnoid hemorrhage: differential effects of alcohol and THC
Source: Neurosurg Rev. 2026 Apr 23;49(1):369. doi: 10.1007/s10143-026-04295-w (PMC13102730; doi:10.1007/s10143-026-04295-w)
Supplement: Supplementary file 1 — Supplementary Material 1 [file 10143_2026_4295_MOESM1_ESM.docx]

**Supplements**

| Parameter | N/mean | %/SD |  |
| --- | --- | --- | --- |
| Age (years) | 56 ±13 years | |  |
| Sex (female) | 674 | 70.6% |  |
| Smoking* | 740 | 78.1% |  |
| Polytox | 31 | 3.2% |  |
| THC | 51 | 5.3% |  |
| Alcohol any | 641 | 67.2% |  |
| Risky Alcohol Use | 44 | 4.6% |  |
| Art. Hypertension** | 549 | 58.7% |  |
| Familial IA*** | 127 | 13.8% |  |
| MIA | 338 | 35.4% |  |
| Sack size (mm) † | 7±5mm | |  |
| Irregular shape IA‡ | 603 | 68.7% |  |
| Multilobular IA§ | 208 | 23.5% |  |
| Post. Circulation IA | 160 | 16.8% |  |
| Ruptured IA | 394 | 41.3% |  |
| WFNS4/5 | 178 | 45.2% |  |
| Modified Fisher 3/4 | 264 | 67.0% |  |
| * Data missing for 7 patients  ** Data missing for 18 patients  *** Data missing for 36 patients  † Data missing for 21 patients  ‡ Data missing for 76 patients  § Data missing for 70 patients | | | |

Supplemental Table 1.: Baseline characteristics of the final study cohort (n=954)

| Parameter | Irregular shape IA | |  | | |
| --- | --- | --- | --- | --- | --- |
|  | yes | no | p | OR | 95%CI |
| Alcohol any | 70.3% | 65.4% | 0.130 | 1.26 | 0.93-1.70 |
| Risky Alcohol Use | 68.4% | 68.7% | 0.972 | 0.99 | 0.49-1.99 |
| Polytoxicomania | 58.6% | 69.0% | 0.235 | 0.64 | 0.30-1.35 |
| THC | 62.5% | 69.0% | 0.342 | 0.75 | 0.41-1.37 |
| Parameter | MIA | |  | | |
|  | yes | no | p | OR | 95%CI |
| Alcohol any | 36.0% | 34.2% | 0.574 | 1.09 | 0.82-1.44 |
| Risky Alcohol Use | 34.1% | 35.5% | 0.849 | 0.94 | 0.50-1.78 |
| Polytoxicomania | 48.4% | 35.0% | 0.125 | 1.74 | 0.85-3.57 |
| THC | 39.2% | 35.2% | 0.561 | 1.19 | 0.67-2.12 |
| Parameter | Post Circulation IA | |  | | |
|  | yes | no | p | OR | 95%CI |
| Alcohol any | 17.2% | 16.0% | 0.645 | 1.09 | 0.76-1.57 |
| Risky Alcohol Use | 22.7% | 16.5% | 0.279 | 1.49 | 0.72-3.08 |
| Polytoxicomania | 6.5% | 17.1% | 0.118 | 0.33 | 0.08-1.41 |
| THC | 11.8% | 17.1% | 0.325 | 0.65 | 0.27-1.55 |

Supplemental Table 2.: Differences in IA characteristics depending of recreational drug use.
